# Effect of dietary iodine on production of iodine enriched eggs

**DOI:** 10.14202/vetworld.2016.554-558

**Published:** 2016-06-04

**Authors:** Shaikh Sumaiya, Sunil Nayak, R. P. S. Baghel, Anju Nayak, C. D. Malapure, Rajesh Kumar

**Affiliations:** 1 Department of Animal Nutrition, College of Veterinary Science and Animal Husbandry, Jabalpur - 482 001, Madhya Pradesh, India; 2 Department of Veterinary Microbiology, College of Veterinary Science and Animal Husbandry, Jabalpur - 482 001, Madhya Pradesh, India

**Keywords:** eggs, iodine, layers, performance

## Abstract

**Aim:**

Objective of this study was to investigate the effect of different levels of iodine supplementation on iodine content of eggs in laying hens.

**Materials and Methods:**

In the experiment, 135 laying hens (White Leghorn) of 55 weeks age were randomly distributed to 5 dietary treatments; each group contained 27 laying hens distributed in three replicates of 9 birds each. Diet T_1_ was control basal layer diet without iodine enrichment in which iodine content (I_2_) was as per NRC recommendation. Basal diets were supplemented with calcium iodate (Ca (IO_3_)_2_) at 5, 10, 15 and 20 mg/kg in T_2_, T_3_, T_4_ and T_5_ groups, respectively. The iodine content in the calcium iodate is 65.21%, therefore, the diets T_2_, T_3_, T_4_ and T_5_ contained 3.25, 6.50, 9.75 and 13.0 ppm iodine, respectively. The laying hens were fed the respective experimental diets ad libitum during the experimental period of 10-week. The iodine content of egg yolk and albumen was analyzed at the end of 5^th^ and 10^th^ week of the experiment. Economics of feeding for the production of iodine enriched egg was calculated at the end of the experiment.

**Results:**

Increasing iodine levels in diet of hens from 0.45 to 13.0 ppm significantly increased egg iodine concentration, the highest concentration of egg iodine was observed in the group fed diet supplemented with 13.0 ppm iodine followed by those fed 9.75, 6.50, 3.25 and 0.45 ppm iodine in diet. There was no significant difference in the iodine levels of unboiled versus boiled eggs. Therefore, the consumers are ensured to receive the optimal levels of iodine from boiled iodine-enriched eggs. Among different diets, minimum and significantly lower feeding cost (Rs. per dozen or per kg eggs) was noticed in hens allotted T_3_ diet (6.50 ppm I_2_). However, feeding cost of hens receiving 3.25 and 9.25 ppm I_2_ was statistically (p<0.05) similar to control group (T_1_). Further, it was noticed that feeding cost (Rs. per dozen or per kg eggs) was significantly increased due to the inclusion of higher level of iodine (13.0 ppm).

**Conclusion:**

It was concluded that supplementing iodine at 6.50 ppm in layers diet was economically better for the production of iodine enriched eggs followed by feed iodine supplementation at 3.25 ppm as compared to control and other treatment groups.

## Introduction

Egg is considered as a complete food with the most of the nutrients required for well-being. In addition to the nutrients already available in the egg if we can alter or incorporate certain health beneficiary nutrients then these eggs will be the choice of food for health conscious people and can reduce the chances of occurrence of certain diseases. Enriched egg with trace minerals results in eggs with superior biological and nutritional value for human consumption [1]. Surveys carried out by Indian Council of Medical Research have shown that no state or union territory is free from the problem of iodine deficiency disorders (IDDs). Out of 586 districts in the country, 281 districts have been surveyed for IDD and 41 districts have been found to be endemic [2], therefore, an improvement of iodine supply is still a great challenge for nutritionists.

Iodine (I_2_) is an essential micronutrient for humans and animals and its deficiency reduces the production of thyroid hormones, leads to the morphological and functional changes of the thyroid gland [3]. Fortification of various nutrients in egg is solely dependent on nutritional manipulation of the laying hen’s ration [4]; therefore, iodine enrichment can be achieved by dietary manipulation of hen’s diet. McDowell [5] pointed to a preferential interception of iodine in the hen ovary and an easy passage of iodine to oocytes. Experimental knowledge of factors participating in the relatively easy iodine passage to yolk makes it possible to increase iodine content in eggs.

The significance of this study is that the iodine supplementation in layer diets could increase the levels of iodine in hen eggs and can lead to prevention of iodine deficiency in humans.

The aim of this study was to investigate the effect of supplementation of iodine at different levels in layers diet on egg iodine content and economics of feeding.

## Materials and Methods

### Ethical approval

The experiment on animals including all procedures of this study was approved by Institutional Animal Ethics Committee.

### Stock, diet and husbandry

In the experiment, 135 laying hens (White Leghorn) of 55 weeks age were randomly distributed to 5 dietary treatments; each group contained 27 laying hens distributed in three replicates of 9 birds each. T_1_ was basal layer diet without iodine enrichment (control), in which iodine (I_2_) content was as per NRC [6] recommendation (0.45 ppm). Basal diets were supplemented with calcium iodate (Ca (IO_3_)_2_) at 5, 10, 15 and 20 mg/kg in T_2_, T_3_, T_4_ and T_5_ groups, respectively. Therefore, dietary iodine (I_2_) content in diets T_2_, T_3_, T_4_ and T_5_ was 3.25, 6.50, 9.75 and 13.00 ppm, respectively. All the diets were formulated as per NRC [6] recommendation using feed ingredients such as maize, soybean meal, and de-oiled rice polish. Ingredients and nutrient composition of all the diets are presented in Table-1. The experiment was for 10 weeks.

**Table-1:** Ingredients and nutrient composition of layer diets (%).

Ingredients	Diets				

	T_1_	T_2_	T_3_	T_4_	T_5_
Maize (kg)	53.00	53.00	53.00	53.00	53.00
DORP (kg)	16.80	16.80	16.80	16.80	16.80
SBM (kg)	19.00	19.00	19.00	19.00	19.00
DCP (kg)	0.400	0.400	0.400	0.400	0.400
LSP (kg)	03.00	03.00	03.00	03.00	03.00
Shell grit (kg)	07.00	07.00	07.00	07.00	07.00
Salt (kg)	0.360	0.360	0.360	0.360	0.360
MnSO_4_ (kg)	0.054	0.054	0.054	0.054	0.054
ZnSO_4_ (kg)	0.10	0.10	0.10	0.10	0.10
CuSO_4_ (kg)	0.01	0.01	0.01	0.01	0.01
FeSO_4_ (kg)	0.139	0.139	0.139	0.139	0.139
Ca (IO_3_)_2_ (kg)	0.004	0.032	0.065	0.097	0.13
Vitamin A, B_2_, D_3_, K (kg)*	0.025	0.025	0.025	0.025	0.025
Vitamin B complex (kg)*	0.005	0.005	0.005	0.005	0.005
Salinomycin (kg)	0.044	0.044	0.044	0.044	0.044
Total (kg)	100.00	100.00	100.00	100.00	100.00
Nutrient composition analyzed					
CP (%)	16.15	16.32	16.20	16.29	16.27
Ca (%)	03.47	03.56	03.38	03.65	03.73
Iodine (mg/kg)	0.45	03.25	06.50	09.75	13.00
Nutrient composition calculated					
ME (kcal/kg)	2602	2602	2602	2602	2602
Lysine (%)	0.66	0.66	0.66	0.66	0.66
Metdionine (%)	0.26	0.26	0.26	0.26	0.26

* Vitamin premix provided (each 250 g contains): Vitamin A – 10,000,000 IU; Vitamin D_3_ – 2,000,000 IU; Vitamin B_1_ – 800 mg; Vitamin B_2_ – 5 g; Vitamin B_6_ - 1.6 g; Vitamin B_12_- 20.5 g; Niacin 12.0 g; Calcium D pantdotdenate - 8.0 g; Vitamin K_3_ - 1.0 g; Vitamin E - 8.0 g; Folic acid – 800 mg, DCP=Digestible crude protein, LSP=Left sacrum posterior, SBM=Soybean meal, DORP=Deoiled rice polish

All the laying hens were shifted to experimental layer house. The system of rearing was cage system. The house was cleaned, white washed, fumigated, and sprayed with disinfectant before introducing the birds in it and was provided with a sufficient light source. Layer mash was offered *ad-libitum* to the hens in feeders. Care was taken that feeders are full of feed at all time and constant watch was kept to avoid feed wastage. An ample supply of clean and fresh drinking water was made available to the laying hens all the time through simple water channel type waterer. During the experiment, the eggs were collected thrice daily, i.e., at 9.00 am; 12.00 noon and at 3.00 pm replicate wise and where weighed at 4.00 pm daily.

### Parameters studied

Total 60 eggs (12 eggs from each treatment) were collected randomly at the end of 5^th^ and 10^th^ week of the experiment for iodine estimation. The iodine concentration in the egg yolk and albumin was determined by a spectrophotometer method (440 nm) using alkaline ashing based on the Sandell-Kolthoff reaction [7]. The principle of the assessment was the reduction of Ce^4+^ to Ce^3+^ in the presence of As^3+^ and the catalytic effect of iodine. Mineralization took place in an alkaline medium at 600°C. In this method, organic sample fades away as the result of a high temperature and iodine released from the organic compound attaches to alcoholic group. The resulting product was not soluble in acid and thus the iodine content was determined chromatographically. In this method, iodine catalyzes a reductive oxidation reaction. In the same manner the iodine content was also measured after boiling the eggs.

All the experimental diets were analyzed for proximate compositions as per AOAC [8]. The economics of feeding was calculated as the cost of feed consumed in rupees for production of one dozen eggs as well as for one kg egg mass.

### Statistical analysis

The data obtained during the experiment were analyzed statistically using the methods described by Snedecor and Cochran [9]. Differences between the treatments were tested for significance by Duncan’s new multiple range test using statistical software SPSS.

## Results

### Effects of dietary iodine on iodine composition of egg

The concentration of iodine in egg yolk and albumen (before and after boiling) of laying hens fed on various levels of iodine after 5^th^ week of supplementation is presented in Table-2. The treatment means of the iodine concentration in eggs (before boiling) of laying hens fed on various levels of iodine after 5^th^ week of supplementation indicated that the inclusion of different levels of iodine significantly (p<0.05) increased iodine content of egg yolk as well as albumen. Maximum and significantly (p<0.05) higher iodine content in egg yolk (1.17±0.025 µg/g) and egg albumin (0.12±0.001 µg/g) were recorded in layers assigned T_5_ (13 ppm I_2_) diet. Iodine content in egg yolk and albumen linearly decreased in layers assigned T_4_ (9.7 ppm I_2_), T_3_ (6.50 ppm I_2_), and T_2_ (3.25 ppm I_2_) diets, respectively. Minimum and significantly (p<0.05) lowest concentration of iodine content in egg yolk (0.19±0.005 µg/g) and egg albumin (0.02±0.002 µg/g) was found in layers assigned T_1_ (control) diet. The treatment means of the iodine concentration in eggs (after boiling) indicated that the use of higher levels of iodine significantly (p<0.05) increased iodine content of egg yolk as well as albumen. Maximum and significantly (p<0.05) higher iodine content of egg (after boiling) was noted in layers assigned T_5_ (13 ppm I_2_) diet. Iodine content in boiled egg yolk and albumen linearly decreased in layers assigned T_4_ (9.75 ppm I_2_), T_3_ (6.50 ppm I_2_) and T_2_ (3.25 ppm I_2_) diets, respectively. It was observed that about 10-15% iodine was lost after boiling the eggs.

**Table-2:** Concentration of iodine in eggs of laying hens fed on various levels of iodine after 5^th^ week of supplementation (before and after boiling).

Treatments	Iodine level (ppm)	Yolk iodine (µg/g)		Albumen iodine (µg/g)	
	
		Before boiling	After boiling	Before boiling	After boiling
T_1_	0.45	0.19^e^±0.005	0.17^d^±0.014	0.02^d^±0.002	0.02^c^±0.002
T_2_	03.25	0.27^d^±0.005	0.20^d^±0.014	0.04^c^±0.001	0.03^c^±0.001
T_3_	06.50	0.52^c^±0.029	0.44^c^±0.015	0.07^b^±0.002	0.06^b^±0.002
T_4_	09.75	0.81^b^±0.008	0.71^b^±0.018	0.08^b^±0.003	0.07^b^±0.001
T_5_	13.00	1.17^a^±0.025	1.03^a^±0.012	0.12^a^±0.001	0.11^a^±0.002

Mean values bearing different superscript differ significantly (p<0.05)

Iodine concentration in egg yolk and albumen (before and after boiling) of laying hens fed various levels of iodine after 10^th^ week of supplementation (before and after boiling) is presented in the Table-3. The trend for iodine content in egg yolk and albumen was similar in the case of both boiled and unboiled eggs. The iodine content of eggs was significantly influenced due to the inclusion of different levels of iodine above recommended levels in the diet of laying hens. Among iodine supplemented groups, maximum and significantly (p<0.05) higher iodine content of egg yolk and albumen was recorded in hens assigned T_5_ (13 ppm I_2_) diet. Iodine content in egg yolk and albumen linearly decreased in layers assigned T_4_, T_3_ and T_2_ (9.75, 6.50 and 3.25 ppm I_2_) diets, respectively. Significantly (p<0.05) lowest iodine concentration in egg yolk and albumin was found in layers assigned T_1_ (control) diet. The treatment means of the iodine concentration indicated that, use of higher levels of iodine significantly (p<0.05) increased iodine content of egg yolk as well as albumen. Further, it was observed that about 10-15% iodine is lost on boiling the eggs.

**Table-3:** Concentration of iodine in eggs of laying hens fed on various levels of iodine after 10^th^ week of supplementation (before and after boiling).

Treatments	Iodine level (ppm)	Yolk iodine (µg/g)		Albumen iodine (µg/g)	
	
		Before boiling	After boiling	Before boiling	After boiling
T_1_	0.45	0.20^e^±0.014	0.18^e^±0.017	0.02^e^±0.001	0.02^e^±0.001
T_2_	03.25	0.28^d^±0.014	0.23^d^±0.020	0.05^d^±0.001	0.04^d^±0.002
T_3_	06.50	0.60^c^±0.014	0.51^c^±0.014	0.07^c^±0.001	0.06^c^±0.002
T_4_	09.75	0.84^b^±0.023	0.72^b^±0.020	0.09^b^±0.002	0.08^b^±0.002
T_5_	13.00	1.20^a^±0.021	1.03^a^±0.002	0.12^a^±0.002	0.11^a^±0.002

Mean values bearing different superscript differ significantly (p<0.05)

### Effect of iodine supplementation on economics of feeding

The economics of feeding for the production of iodine enriched eggs is presented in Table-4. The results of this study revealed that among iodine supplemented diets, significantly (p<0.05) lowest feeding cost (Rs. per dozen or per kg egg mass) was noticed in layers receiving 6.50 ppm iodine in their diet. However, feeding cost (Rs. per dozen or per kg egg mass) of layers receiving 3.25 and 9.75 ppm iodine in their diet was statistically similar (p>0.05) and comparable to control (0.45 ppm I_2_). Further, inclusion of higher levels of iodine significantly (p<0.05) increased the feeding cost (Rs. per dozen or per kg eggs) in layers. Feed cost (Rs. per kg) was near about similar in all treatments since the price of iodine source was very low.

**Table-4:** Economics of feeding for production of iodine enriched eggs.

Treatments	Iodine level (ppm)	Feed cost kg feed/dozen eggs (Rs.)	Feed cost kg feed/kg eggs (Rs.)
T_1_	0.45	32.56^b^±0.167	46.22^b^±0.763
T_2_	03.25	32.43^b^±0.167	46.31^b^±0.480
T_3_	06.50	32.06^c^±0.063	45.58^c^±0.184
T_4_	09.75	32.81^b^±0.418	46.80^b^±0.663
T_5_	13.00	34.07^a^±0.126	50.47^a^±0.223

Mean values bearing different superscript differ significantly (p<0.05)

## Discussion

Iodine content in eggs yolk and albumen, both after 5^th^ week and 10^th^ week of supplementation, linearly increased with the increasing iodine levels (0.45, 3.25, 6.50 and 13.0 ppm I_2_) in their diets. This increment may be due to the higher iodine transfer from feed to egg. The results of iodine concentration are consistent with the findings of Kaufmann *et al*. [10], who found a significant linear correlation (r=0.93) between iodine content in feed mixture up to 5 mg/kg and iodine content in yolk. Yang *et al*. [11] indicated that laying hens might be a good carrier for transporting iodine, from diet to egg. Ramune *et al*. [12] found that in eggs from laying hens receiving feed containing 1 and 4 ppm iodine, egg iodine content was 24% and 196 % more than control group. Moreover, Songserm *et al*. [13] revealed that 4000 mg supplemental iodine/ton diet in two forms potassium iodide or potassium iodate increased the iodine concentration of eggs. In support to the results of this study introduction of iodine supplement from 1 to 5 mg/kg into the diet of hens enabled the significant enrichment of the egg iodine content [14]. Saki *et al*. [15] indicated that increased albumen and yolk iodine is proportional to level of supplementary iodine in diet (p<0.05). Similarly, Gjorgovska and Kiril [16] stated that when laying hen feed enriched with 5 mg iodine/kg diet, the yolk from eggs of such hens is better source of iodine and can fulfill 11-15% of daily requirements for adult people if they consume one egg per day. Travnicek *et al*. [17] found that when iodine content in feed mixtures was raised from 0.22 to 1.18 mg/kg dietary dry matter, iodine content in egg yolk was increased significantly. Malak *et al*. [18] in their experiment also observed that increasing iodine levels from 0.3 to 9.6 ppm in hens diet significantly increased egg iodine concentration and the highest concentration of egg iodine was observed for the hens fed diet supplemented with 9.6 ppm followed by those fed 4.8 ppm iodine, while these high levels of iodine negatively affected the egg production. They concluded that supplementation of laying hen diet with 2.4 mg iodine/kg diet gave iodine enriched egg which can fulfill 44% of daily requirements of iodine for children of 1-10 years. The results of this study regarding egg iodine concentration are also in concurrent with the findings of Rottger *et al*. [19] who recorded linear correlation between iodine content in feed and egg yolk.

In this study, the reduction in the iodine content of eggs after boiling ranged between 10% and 15%. In concurrent to this lower iodine recovery in the case of egg boiled for 30 min was also reported by Lipiec *et al*. [20]. While, Songserm *et al*. [13] studied iodine stabilization of iodine-enriched eggs before boiling followed by two methods of boiling, i.e. short time (soft boiled egg) and long time (hardboiled egg) and reported no any significant (p>0.05) difference in the iodine levels of boiled and unboiled eggs.

Among iodine supplemented diets, minimum and significantly (p<0.05) lowest feeding cost (Rs per dozen or per kg egg mass) was noticed in layers receiving 6.50 mg/kg iodine in their diet. Further, the inclusion of higher levels of iodine significantly (p<0.05) increased the feeding cost in layers. In support to our results, Malak *et al*. [18] also reported improved economic efficiency of laying hens fed 0.6, 1.2, 2.4 and 4.8 ppm iodine in their diet as compared to control.

## Conclusion

From the results of the present experiment, it was concluded that iodine at 6.50 ppm in layers diet was economically better for the production of iodine enriched eggs followed by feed iodine supplementation at 3.25 ppm as compared to control and other treatment groups.

## Authors’ Contributions

SN and RPSB have designed the plan of work. SS carried out the laboratory work and analyzed the results. AN and CDM drafted and RK revised the manuscript. All the authors read and approved the final manuscript.
